# Differences in reported linguistic thermal sensation between Bangla and Japanese speakers

**DOI:** 10.1186/s40101-017-0139-5

**Published:** 2017-06-05

**Authors:** Aklima Khatun, Md. Abdul Hasib, Hisaho Nagano, Akihiro Taimura

**Affiliations:** 1 Graduate school of Fisheries and Environmental Sciences, Nagasaki University, 1-14 Bunkyo-machi, Nagasaki, 852-8521 Japan; 2 Graduate School of Engineering, Nagasaki University, 1-14 Bunkyo-machi, Nagasaki, 852-8521 Japan

**Keywords:** Thermal sensation, Thermal comfort, Linguistic expression, Ethnicity

## Abstract

**Background:**

Thermal sensation is a fundamental variable used to determine thermal comfort and is most frequently evaluated through the use of subjective reports in the field of environmental physiology. However, there has been little study of the relationship between the semantics of the words used to describe thermal sensation and the climatic background. The present study investigates the linguistic differences in thermal reports from native speakers of Bangla and Japanese.

**Methods:**

A total of 1141 university students (932 in Bangladesh and 209 in Japan) responded to a questionnaire survey consisting of 20 questions. Group differences between Bangladeshi and Japanese respondents were then tested with a chi-square test in a crosstab analysis using SPSS (version 21).

**Results:**

For the Bangla-speaking respondents, the closest feeling of thermal comfort was “neutral” (66.6%) followed by “slightly cool” (10.2%), “slightly cold” (6.0%), “slightly hot” (4.1%), and “cold” (3.8%). For the Japanese respondents, the closest feeling of thermal comfort was “cool” (38.3%) followed by “slightly cool” (20.4%), “neutral” (14.6%), “slightly warm” (13.1%), and “warm” (10.7%).

Of the Bangladeshi respondents, 37.7% reported that they were sensitive to cold weather and 18.1% reported that they were sensitive to hot weather. Of the Japanese respondents, 20.6% reported that they were sensitive to cold weather and 29.2% reported that they were sensitive to hot weather. Of the Bangladeshi respondents, 51.4% chose “higher than 29 °C” as hot weather and 38.7% of the Japanese respondents chose “higher than 32 °C” as hot weather. In the case of cold weather, 43.1% of the Bangladeshi respondents selected “lower than 15 °C” as cold weather and 53.4% of the Japanese respondents selected “lower than 10 °C” as cold weather.

**Conclusions:**

Most of the Bangla-speaking respondents chose “neutral” as the most comfortable temperature, and most of the Japanese respondents chose “cool.” Most of the Bangladeshi respondents reported that they were sensitive to “cold temperatures,” but most of the Japanese respondents reported that they were sensitive to “hot temperatures.”

## Background

Thermal sensation is a fundamental variable used to determine thermal comfort, which is a major determinant driving behavioral thermoregulation. Thermal comfort affects the state of mind, which expresses satisfaction with the thermal environment. Thermal sensation is perceived through the brain and it is universal for humans irrespective of nationality, while perceived thermal sensation expressed by language is not universal and varies significantly between individuals and across cultures [1]. Thermal sensation and comfort level are not the same in all climatic areas; perceptions and linguistic expressions also vary from culture to culture.

Few studies have been conducted to identify perceived thermal sensation and comfort using a questionnaire survey. Tochihara explores the evidence of heat acclimatization in the words that Indonesian and Japanese native speakers use to express thermal sensation [2]. Lee’s study examines the linguistic dimensions of the expressions “warm” and “slightly hot” in Korean speakers [3]. Another of Lee’s studies discusses particular linguistic elements within thermal sensation descriptors used by Japanese and Koreans compared with the ISO scale in English [1].

Human environmental adaptability is influenced by a variety of factors [4]; thermal comfort depends on a combination of external (environmental: air temperature, relative humidity, air velocity) and internal (personal: metabolic rate and clothing) parameters. People in different regions may have different thermal sensations or preferences even under the same climatic conditions [5, 6]. The effects of excessive cold and heat occur immediately, but the long-term impact of exposure to cold lasts longer [7].

Physiological and linguistic heat acclimatization are not the same. Physiological heat acclimatization is a physical response within the human body, whereas linguistic heat acclimatization is projected onto the human mind as a preference through words. The linguistic elements in heat acclimatization are encoded in one’s own language through inter-subjective communication [2]. The human mind as a multi-leveled process is dependent on the interactions between the mind, body, environment, and culture, and different linguistic dimensions may cause a bias in interpreting the relationship between thermal sensation and environmental temperatures [1]. Generalizing and comparing cross-linguistic expressions of thermal sensation and comfort level is therefore complex. Subjective perceptions, such as thermal sensation and discomfort, function as behavioral controllers. Most people subjectively understand their own heat or cold tolerance, which is related to behavioral adjustment, physiological and psychological acclimatization, and habituation [8].

In this study, two different climate regions were chosen for comparison of linguistic thermal sensation and comfort: Bangladesh, which falls in the tropical zone, and Japan, which falls in the temperate zone. We selected Bangladesh because it is one of the most densely populated countries in the world, and coastal regions of Bangladesh are vulnerable to the effects of climate change. More than 33% of the world’s population lives in the humid tropics, which is characterized by consistently high monthly temperatures and rainfall that exceeds evapotranspiration on most days of the year. Bangladesh has a tropical monsoon climate, with a mild winter and a hot, humid summer for most of the year. The annual rainfall is high, the average temperature is 26.1 °C, and the relative humidity is 65.8%; the highest annual temperature is 42.4 °C, and the lowest is 7.2 °C [9]. Japan is located in the temperate zone. The annual temperature is 16.7 °C, and the relative humidity is 72.2%; the highest annual temperature is 31.3 °C, and the lowest is 3.1 °C [9].

Bangladesh’s official language Bangla is one of the most widely spoken languages in the world, and Japanese language is also like that. As linguistic differences between cultures may cause confusion when interpreting thermal perceptions measured by different languages [1], it is important to quantify the characteristics of individuals’ thermal comfort in regions with different climatic conditions. For example, people living in areas with a hot climate may be better adapted to hot weather and tolerate higher thermal conditions than those who are usually exposed to temperate climates [10]. This study used questionnaires to assess the comfort and sensation of physical and psychological parameters of Bangla and Japanese native speakers. This questionnaire also assessed the relationship between cultural, social, perception and psychological aspects.

Bangla contains distinct linguistic distinctions corresponding to each point of the ASHRAE (American Society of Heating, Refrigerating and Air-Conditioning Engineers) 11-point thermal scale. Bangla uses the term “ushno” (warm) to describe comfortable situations, indicating not only temperature but also other aspects of comfort. In this sense, it functions similarly to the Japanese word “suzushi” (cool), which can also be used to describe non-thermal aspects of comfort. The contrast between “ushno” and “suzushi” suggests that baseline notions of thermal comfort may be different for individuals in these two countries.

Such contrasts are the focus of the present study, which compares linguistic reports of thermal sensation among Bangla and Japanese speakers. Our hypothesis is that the Bangladeshi respondents’ most comfortable temperature descriptor would be “warm” given that most of the year in Bangladesh is very hot, and thus, these speakers would be less cold-tolerant [11], and that, for Japanese respondents, the most comfortable temperature descriptor would be “cool” because the summer in Japan is shorter, and these speakers’ heat tolerance would be lower than their cold tolerance [12].

## Methods

A total of 1141 university students responded to a survey questionnaire consisting of 20 questions. There were 932 (615 males and 316 females) BD respondents (mean age, 21.6 ± 2.4 year) and 209 (81 males and 128 females) JP respondents (mean age, 19.9 ± 1.8 year). We collected data in Bangladesh (BD) from December 2014 to January 2015 and in Japan (JP) in two surveys, July 2014 (1st survey, 98 respondents) and December 2015 (2nd survey, 111 respondents). Each respondent was independent, with no overlap. The BD and JP respondents had the same thermal temperature experience as other individuals within their country, meaning direct exposure to cold and hot weather. The Ethics Committee of the Faculty of Environmental Science, Nagasaki University, approved this survey protocol. The survey respondents were verbally informed of the aims and benefits of this survey, and informed consent was secured from all respondents before the questionnaire survey data was collected. We obtained these data from one university in Japan and three universities in Bangladesh (Dhaka, Rajshahi, and Khulna) (university A, 290 students; university B, 456 students; university C, 187 students; the male to female ratio was 174:113, 263:192, and 174:10, respectively).

## Questionnaire

The questionnaire was presented in each native language (Bangla and Japanese) and consisted of a total of 20 questions. This questionnaire had three sections.

- ○ Thermal sensation and comfort–15 questions (taken from Tochihara et al. [2])
- ○ Daily heating and cooling habits–2 questions (taken from Tochihara et al. [2])
- ○ Daily activities–3 questions
  - > Activity level of the day
  - > Breakfast frequency
  - > Sleeping time

Fifteen questions concerned thermal sensation and comfort, and two questions concerned daily heating and cooling habits. These 17 questions were taken from Tochihara and Lee’s study [2]. Another three questions concerned respondents’ daily activities (activity level of the day, frequency of eating breakfast, and amount of time spent sleeping each day).

An 11-point thermal scale was used to measure thermal sensation. Typically, scales used to measure thermal sensation have been formatted as categorical scales using the points cold, cool, slightly cool, neutral, slightly warm, warm, and hot (ASHRAE 1992) [3]. It is reasonable to assume that there will be variable linguistic dimensions in thermal sensation descriptors among different linguistic groups because there are a variety of climates on Earth. ISO 10551 (1995) pointed out that bias would result from the vocabulary choices in each language when using thermal sensation scales.

A number of subjective scales have been developed for surveys to investigate thermal comfort, primarily using categorical scales and evaluating thermal sensation based on human physiology and ergonomics [4]. In this study, we used the ASHRAE 11-point thermal sensation scale [13], which assumes that different words have different linguistic dimensions and that one cannot assume that intervals between adjacent descriptors are identical cross-linguistically. Other non-English researchers may have similar difficulties when adapting the ISO scale into their own language [3]. Table 1 shows the descriptive terms used in the English-based ISO scale and the corresponding terms in Bangla and Japanese that were used in the questionnaire. Although similar terms of thermal sensation can be found in each of these languages, their exact semantic territory does not perfectly correspond. This imperfect correspondence is to be expected, given that relatively few mental components are so fundamental that they cannot be shaped through socialization and cultural participation, and hence, manifest differently cross-linguistically [14].

**Table 1:**
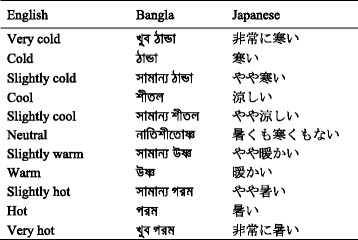
Corresponding temperature terms in English, Bangla, and Japanese used in the questionnaire

## Data analysis

First, the frequency distribution of responses and their percentages for each question were analyzed. Group differences between Bangladeshi and Japanese respondents were then tested with a chi-square test in a crosstab analysis using SPSS (version 21).

## Results

We collected data from Japanese (JP) respondents at two different times of the year. The most frequent description of hot weather as “higher than 29 °C” was 52.0 and 50.5% by respondents of the 1st and 2nd Japanese surveys, respectively; 22.4% chose “higher than 20 °C” in the 1st survey, but only 17.1% chose “higher than 20 °C” in the 2nd survey; 21.4% chose “higher than 32 °C” in the 1st survey, and 28.8% chose “higher than 32 °C” in the 2nd survey. For cold weather, 52.0 and 54.1% of respondents chose “lower than 10 °C” in the 1st and 2nd surveys, respectively; 17.3 and 32.4% of respondents chose “lower than 15 °C” as cold weather in the 1st and 2nd surveys, respectively; and in the 1^st^ survey, 14.3% of respondents chose 20 °C as the threshold for cold weather, but in the 2nd survey, only 4.5% of respondents chose 20 °C as cold weather. There were no significant differences between the results of the two times of the surveys. We therefore combined the JP respondents’ results and compared them with the BD respondents’ results. Group differences between BD and JP for questions 1 to 17 of *P* < 0.001 were considered statistically significant.

Question a. How active are you from day to day?

Among BD and JP respondents, 45.8 and 33.8% spend a large part of the day in a sitting position performing sedentary activities (desk work, watching television); 44.3 and 50.0% of respondents not only had a static lifestyle but also performed light sports and housework; 6.9 and 13.2% of respondents performed jobs involving long durations of standing or moving about regularly exercised; 3.0 and 2.9% of respondents exercised almost every day; BD and JP respondents respectively. A group difference of *P* < 0.001 was considered statistically significant.

Question b. How often do you eat breakfast?

Among BD and JP respondents, 2.8 and 22.4%, respectively, seldom ate breakfast; 8.2 and 15.1% of respondents ate breakfast twice a week; 19.5 and 14.1% of respondents ate breakfast 4–5 times a week; 69.5 and 48.3% of respondents ate breakfast almost every day. A group difference of *P* < 0.001 was considered statistically significant.

Question c. How long do you sleep every day?

Among BD and JP respondents, 11.1 and 36.7%, respectively, slept less than 6 h; 44.0 and 49.8% of respondents slept 6–7 h; 30.7 and 11.1% of respondents slept 7–8 h, and 14.1 and 2.4% of respondents slept 8 h or more. A group difference of *P* < 0.001 was considered statistically significant.

Question 1. What thermal sensations do you feel as the most comfortable?

**Fig. 1:**
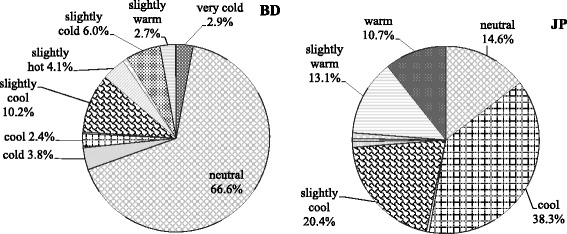
Most comfortable thermal sensation of Bangladeshi (BD) and Japanese (JP) respondents

For BD respondents, the closest feeling of thermal comfort was “neutral” (66.6%) followed by “slightly cool” (10.2%), “slightly cold” (6.0%), “slightly hot” (4.1%), and “cold” (3.8%), while for JP respondents, the closest feeling of thermal comfort was “cool” (38.3%) followed by “slightly cool” (20.4%), “neutral” (14.6%), “slightly warm” (13.1%), and “warm” (10.7%) (Fig. 1).

Question 2. Does your home have a cooling system?

Most of the BD and JP respondents had a cooling system. Only 3.0 and 2.9% of BD and JP respondents had no cooling system in their homes, respectively. Only electric fans were considered in our definition of cooling system.

Question 3. Does your home have a heating system?

In Bangladesh, few people used an electric room heater (5.4%) or air conditioner for warming (3.0%). Among JP respondents, 97.1% had an air conditioner and room heater in their homes.

Question 4. To which weather conditions are you most sensitive?

**Fig. 2:**
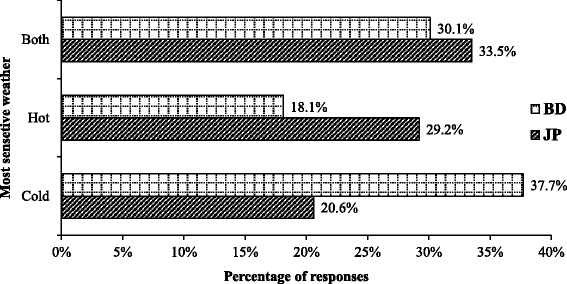
Weather to which Bangladeshi (BD) and Japanese (JP) respondents were most sensitive

Among BD and JP respondents, 37.7 and 20.6%, respectively, reported being sensitive to cold, 18.1 and 29.2% of respondents reported being sensitive to heat, and 30.1 and 33.5% of respondents reported being sensitive to both conditions (Fig. 2).

Questions 5 to 12 required an interpretation based on the concept of conditional probability. Results are summarized in Table 2, where N means neutral, S satisfied, DS dissatisfied, W warm, H hot, VH very hot, SH slightly hot, SW slightly warm, SCD slightly cold, SCL slightly cool, CD cold, and CL cool. For example, under the result for question 6, S/W indicates that 66.3% of JP and 41.0% of BD respondents felt satisfied in warm conditions; DS/W indicates 5.8% of JP, and 35.6% of BD respondents felt dissatisfied in warm conditions.

**Table 2:** Summary of responses to questions 5–12 in the questionnaire

Question no.		Japanese	Bangladeshi	*χ* ^2^	*P* value
5	P (S/SH) P (DS/SH)	3.4 79.2	55.0 21.0	290.902	<0.001
6	P (S/W) P (DS/W)	66.3 5.8	41.0 35.6	89.837	<0.001
7	P (S/SCD) P (DS/SCD)	8.7 63.9	57.1 21.1	208.674	<0.001
8	P (S/CL) P (DS/CL)	83.6 1.0	26.7 48.3	256.669	<0.001
9	P (SH/H) in hot weather P (N/VH) in hot weather	49.5/32.7 2.9/5.8	22.5/21.7 12.4/12.2	188.102	<0.001
10	P (W/N) in cold weather P (SH/SW) in cold weather	72.1/5.3 1.9/10.6	16.5/17.8 12.8/12.4	279.343	<0.001
11	P (CD/SCD) in cold weather P (CL/SCL) in cold weather	53.8/29.8 2.4/1.0	27.1/10.1 17.9/15.7	157.999	<0.001
12	P (CL/CD) in hot weather P (N/SCL) in hot weather	85.1/1.4 1.9/3.8	23.4/9.6 17.4/16.4	279.278	<0.001

In question 5, 55.0% of BD respondents were satisfied with a “slightly hot” feeling. In contrast, 79.2% of JP respondents were dissatisfied with a “slightly hot” feeling.

In question 6, 41.0% of BD respondents were satisfied and 35.6% were dissatisfied with a “warm” feeling. Among JP respondents, 66.3 and 5.8% were satisfied and dissatisfied, respectively.

In question 7, 57.1% of BD respondents were satisfied with a “slightly cold” feeling. Among JP respondents, 63.9% were dissatisfied with the same feeling.

In question 8, 48.3% of BD respondents were dissatisfied in “cool” and 83.6% of JP respondents were satisfied in the same condition.

For question 9, respondents were presented with the following scenario. Imagine it is a hot and humid summer. You walk out from an air-conditioned building to the hot and humid outside. At that moment, you feel “a little thermally uncomfortable” but you do not yet sweat. Your thermal feeling would be?

Among BD respondents, 22.5 and 21.7% felt “slightly hot” and “hot,” respectively, whereas JP respondents 49.5 and 32.7% felt “slightly hot” and “hot,” respectively.

Question 10 presented a different scenario. Imagine it is a cold and windy winter. You walk into a well-heated building from the cold and windy outside. You feel “thermally comfortable” without any shivering. Your thermal feeling would be?

Among BD respondents, 16.5 and 17.8% felt “warm” and “neutral,” respectively, whereas in JP respondents, 72.1 and 5.3% reported “warm” and “neutral,” respectively.

Question 11. Imagine it is a cold and windy winter. After staying in a well-heated building, You then walk out from the well-heated building to the cold outside. Outdoors, you feel “a little thermally uncomfortable” with some goose bumps, but you do not shiver. Your thermal feeling would be?

Among BD respondents, 27.1 and 10.1% felt “cold” and “slightly cold,” respectively, whereas JP respondents, 53.8 and 29.8% reported “cold” and “slightly cold,” respectively.

Question 12. Imagine it is a hot and humid summer. After walking in the street without any shade and experiencing some sweating, you then walk into an air-conditioned building. Inside the building, you feel “thermally comfortable.” At this moment, your thermal feeling would be?

Among 23.4 and 9.6% of BD respondents felt “cool” and “cold,” respectively, whereas JP respondents, 85.1 and 1.4% reported “cool” and “cold,” respectively.

Question 13. What word describes the feeling between “neutral” and “cold”?

In this case, 40.8 and 55.8% of BD and JP respondents, respectively, chose “slightly cool”; 28.1 and 19.2% chose “cool,” and 29.8 and 25.0% chose “slightly cold.”

Question 14. What word(s) describes the feeling between “neutral” and “hot”?

To this, 43.5 and 49.5% of BD and JP respondents chose “slightly hot,” 34.5 and 20.2% chose “slightly warm,” and 21.8 and 30.3% of BD and JP chose “warm.”

Question 15. What word is the opposite of “warm”?

To this, 72.8% of BD respondents and 73.1% of JP respondents answered that “cool” is opposite; 15.8% of BD and 19.2% of JP respondents answered “cold”; 6.9% of BD and 7.7% of JP respondents answered “slightly cold.”

Question 16. What temperature do you consider to be “hot weather”?

**Fig. 3:**
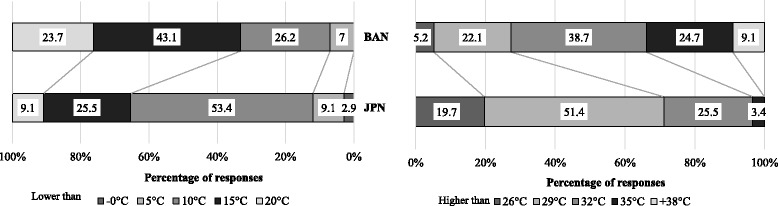
The temperature that Bangladeshi (BD) and Japanese (JP) respondents considered as “hot weather” and “cold weather”

A total of 38.7% of BD and 25.5% of JP respondents selected “higher than 32 °C” as hot weather. A total of 22.1% of BD and 51.4% of JP respondents selected “higher than 29 °C” as hot weather. Higher than 26, 35, 38, and 40 °C were selected as hot weather by 5.2 and 19.7, 24.7 and 3.4, 6.4 and 0.0, and 2.9 and 0.0% of BD and JP respondents, respectively (Fig. 3).

Question 17. What temperature do you consider to be “cold weather”?

A total of 43.1% of BD and 25.5% of JP respondents selected “lower than 15 °C” as cold weather. A total of 26.2% of BD respondents and 53.4% of JP respondents selected “lower than 10 °C” as cold weather. Lower than −5, 0, 5, and 20 °C were selected as cold weather by 0.0 and 1.0, 0.0 and 1.9, 7.0 and 9.1, and 23.7 and 9.1% of BD and JP respondents, respectively (Fig. 3).

## Discussion

The purpose of this study was to investigate the linguistic differences in reports of thermal sensation between Bangla and Japanese native speakers. We found that the linguistic expression of temperature for speakers in these climatic and cultural regions showed several interesting contrasts. Not only did the temperature they reported as most comfortable differ, they also offered different judgments about the relative level of thermal sensation under various scenarios. As such, this study may provide evidence about the thermal neutral zone as formulated in the minds of speakers. This study used the ASHRAE thermal sensation scale, which has previously been shown to be unstable under translation into five languages (English, French, Greek, Portuguese, and Swedish) [15].

What are the thermal sensation and comfort differences between BD and JP respondents?

Hot and humid weather makes people tired more quickly than cold weather, and if the body temperature rises above 38 °C and below −10 °C, physical and cognitive functions are impaired. If humidity and temperature are lower, productivity increases. High temperatures affect people’s skill level, which leads to a decline in capacity and productivity [16]. The temperature in Bangladesh in the summer is more than 38 °C. The physical characteristics (height, weight, age) of the BD and JP respondents were the same, but according to responses about daily activities, 45.8% of BD respondents had a sedentary lifestyle, whereas 33.8% of JP respondents had a sedentary lifestyle. The JP respondents were more active. Moreover, 29.2% of JP respondents were sensitive to “hot weather” and 37.7% of the BD respondents were sensitive to “cold weather.”

In question 4, the most comfortable thermal sensation of the BD respondents was “neutral” (66.6%), whereas among JP respondents, only 14.6% chose “neutral.” The most comfortable thermal sensation of JP respondents was “cool” (38.3%), whereas among BD respondents, only 2.4% chose “cool.” The next comfortable thermal sensation was “slightly cool” for both BD and JP respondents, 10.2 and 20.4%, respectively.

Bangladesh has a hot climate, and for this reason, people in this area may have a lower tolerance to cold and do not prefer cold temperatures. On the other hand, Japan has a colder climate and for this reason, people in this area have a lower tolerance to heat and consider summer to be intolerable. Interestingly, [2] found that for Indonesians, the most comfortable thermal sensation was “cool” (75%). In Indonesia, the temperature is almost constant throughout the year, with an average temperature of 27.8 °C [17].

Question 5 also found a difference between BD and JP groups. In particular, 55.0% of BD respondents were satisfied in “slightly hot” conditions, but only 3.4% of JP respondents were satisfied and 79.2% were dissatisfied in such conditions. Similarly, in question 8, 83.6% of JP respondents were satisfied in “cool” thermal conditions, but only 27.7% of BD respondents were satisfied. In fact, 48.3% of BD respondents were dissatisfied in ‘cool’ conditions. In questions 7 and 8, Bangladeshi respondents preferred “slightly cold” and “slightly hot” to “cool” and “warm,” respectively. In practice, however, it is very difficult for people to detect a difference between “slightly cold” and “cool” or “slightly hot” and “warm.” Bangla has separate words for these conditions, but their meanings are very close.

To clarify the differences between BD and JP respondents’ reports of “satisfied” and “dissatisfied” in questions 5 to 8, a discriminant analysis was applied. The standardized canonical discriminant function coefficients were −0.354, 0.377, −0.682, and 0.759 for questions 5 to 8, respectively. The canonical correlation was 0.422 (*P* < 0.001). As a result, 78.7% of the original grouped cases were correctly classified. BD respondents were satisfied in “slightly hot” and “slightly cold” conditions, and JP respondents were satisfied in “warm” and “cold” conditions. This result suggests that the concept of conditional probability accounts for the large differences observed between BD and JP respondents.

Global warming countermeasures in each area of the world may vary depending on weather patterns, local customs, and cultural norms. The study found that 97.1% of JP respondents had air conditioning, but very few BD respondents used either air conditioning (3.0%) or a heating system (5.4%) in their homes. The Japanese Ministry of the Environment conducted a campaign in 2005 to encourage citizens to reduce electricity consumption by limiting their use of air conditioning. The Ministry recommended that people maintain a temperature of 28 °C in summer and 20 °C in winter. As a result of this campaign, the Japanese became more conscious about how their comfort corresponded to room temperature. The Bangladeshis, on the other hand, appear to be less concerned with the impact of temperature on their personal comfort, possibly due to the lack of means of controlling it.

In question 10, 72.1% of JP respondents selected “warm,” but only 16.5% of BD respondents selected “warm” in replay to this question. It seems that the different ethnicities had a different choice for their thermal comfort level. The fact that the Japanese and Bangla languages do not share a known common linguistic ancestor may account for some of these discrepancies.

In question 16, JP respondents chose “higher than 29 °C” (51.4%) and BD respondents chose “higher than 32 °C” (38.7%) as hot weather. It seems Bangladeshis, who are native to a tropical climate, have a higher heat tolerance than the Japanese, who are native to a temperate climate [18]. In question 17, JP respondents chose “lower than 10 °C” (53.4%) as cold weather and BD respondents chose “lower than 15 °C” (43.1%) as cold weather. This response aligns with the findings in [11], which found that the Japanese have a higher local cold tolerance than Bangladeshis as evaluated by a cold-induced vasodilation test. To the same question, Indonesian respondents chose “hot” weather as “higher than 32 °C” (30.0%) and chose “cold” weather as “lower than 20 °C” (55.0%) [2]. Indonesian respondents therefore held similar judgments about heat as did BD respondents but different responses with respect to cold. This may be due to the fact that Indonesian respondents lacked direct exposure to cold weather [2]. Indonesia has a middle tropical climate, whereas Bangladesh has a north tropical climate, and therefore, more Bangladeshis have directly experienced cold weather. Japanese natives have even greater acquaintance with cold weather, as Japan lies in the temperate zone. The thermal sensation reported by Indonesians, Bangladeshis, and Japanese thus showed a gradual variation reflecting adaptation to different local levels of heat and cold. Natives from tropical and temperate regions judge the relative temperature differently with correspondingly different judgments in comfort levels.

## Conclusions

The purpose of this study was to investigate the linguistic differences between Bangla and Japanese speakers with respect to reports of thermal sensation. Most of the Bangla-speaking respondents chose “neutral” as the most comfortable temperature, whereas most Japanese respondents chose “cool.” Most Bangladeshi respondents reported that they were sensitive to “cold temperatures,” whereas most Japanese respondents reported they were sensitive to “hot temperatures.” These differences arguably reflect the linguistic thermal acclimatization effects of living in different climates.
